# Progression of functional and structural glaucomatous damage in relation to diurnal and nocturnal dips in mean arterial pressure

**DOI:** 10.3389/fcvm.2022.1024044

**Published:** 2022-11-15

**Authors:** Jesus D. Melgarejo, Jan Van Eijgen, Dongmei Wei, Gladys E. Maestre, Lama A. Al-Aswad, Chia-Te Liao, Luis J. Mena, Thomas Vanassche, Stefan Janssens, Peter Verhamme, Karel Van Keer, Ingeborg Stalmans, Zhen-Yu Zhang

**Affiliations:** ^1^Research Unit Hypertension and Cardiovascular Epidemiology, KU Leuven Department of Cardiovascular Sciences, Studies Coordinating Centre, Katholieke Universiteit Leuven, Leuven, Belgium; ^2^Laboratory of Neurosciences, Faculty of Medicine, University of Zulia, Maracaibo, Venezuela; ^3^Department of Ophthalmology, University Hospitals UZ Leuven, Leuven, Belgium; ^4^Research Group of Ophthalmology, Department of Neurosciences, Katholieke Universiteit Leuven, Leuven, Belgium; ^5^Rio Grande Valley Alzheimer’s Disease Resource Center for Minority Aging Research (RGV AD-RCMAR), University of Texas Rio Grande Valley, Brownsville, TX, United States; ^6^School of Medicine, Institute for Neuroscience, University of Texas Rio Grande Valley, Harlingen, TX, United States; ^7^Department of Human Genetics, School of Medicine, University of Texas Rio Grande Valley, Brownsville, TX, United States; ^8^Department of Ophthalmology, New York University (NYU) Grossman School of Medicine, NYU Langone Health, New York, NY, United States; ^9^Department of Informatics, Universidad Politécnica de Sinaloa, Mazatlán, Mexico; ^10^KU Leuven Department of Cardiovascular Sciences, Centre for Molecular and Vascular Biology, Katholieke Universiteit Leuven, Leuven, Belgium; ^11^Division of Cardiology, Department of Internal Medicine, University Hospitals UZ Leuven, Leuven, Belgium

**Keywords:** ambulatory blood pressure monitoring (ABPM), diurnal MAP profile, nocturnal hypotension, glaucoma progression, primary open-angle glaucoma

## Abstract

**Background:**

Systemic hypoperfusion plays a pivotal role in the pathogenesis of primary open-angle glaucoma (POAG). Extreme dips in mean arterial pressure (MAP) due to high 24-h variability are associated with POAG, however, whether this is driven by diurnal or nocturnal dips remains undocumented. We aimed this study to investigate the association of POAG damage with variability and dips in the diurnal and nocturnal MAP.

**Methods:**

We conducted a retrospective longitudinal study that included 110 POAG patients who underwent 24-h ambulatory blood pressure monitoring. Our outcomes included (i) functional [visual field defects expressed as mean deviation (MD)] and (ii) structural (optic disc cupping obtained from cup-to-disc ratio) glaucoma damage. MAP variability independent of the mean (VIMmap) was computed for diurnal and nocturnal MAP. Dips were the five diurnal and three nocturnal lowest drops in MAP. We also calculated the night-to-day ratio. We applied mixed models to evaluate the progression of visual field defects and optic disc cupping in relation to diurnal and nocturnal MAP measures.

**Results:**

The mean age was 64.0 y (53% women). The median follow-up was 9 years. In adjusted mixed models, functional progression of glaucoma damage was associated with VIMmap (−2.57 dB change in MD per every 3 mmHg increase in VIMmap; *P* < 0.001) and diurnal MAP dips (changes in the MD ranged from −2.56 to −3.19 dB; *P* < 0.001). Every 5 mmHg decrease in the nocturnal MAP level was associated with −1.14 dB changes in MD [95% confidence interval (CI), −1.90 to −0.40] and 0.01 larger optic disc cupping (95% CI, 0.01–0.02). Lower night-to-day ratio was also related to both outcomes (*P* ≤ 0.012). Functional glaucoma damage worsened if nocturnal hypotension was combined with high variability or extreme dips in the diurnal MAP (*P* ≤ 0.022).

**Conclusion:**

Progression of glaucoma damage in POAG associates with high variability and extreme dips in the diurnal MAP. Structural glaucoma damage seems more vulnerable to nocturnal hypotension. Ambulatory blood pressure monitoring allows the assessment of sporadic diurnal and persistent nocturnal hypotension episodes. These phenotypes might offer an opportunity to improve the risk-stratification of open-angle glaucoma (OAG).

## Introduction

Open-angle glaucoma (OAG) is a progressive optic neuropathy characterized by loss of retinal ganglion cells and visual deterioration in eyes with or without high intraocular pressure (1). The pathogenesis of OAG is unclear but recent evidence focuses on risk factors that affect the ocular perfusion pressure (2, 3), with nocturnal hypotension being the most important systemic vascular risk factor (4–6). The most plausible hypothesis is that low mean arterial pressure (MAP) leads to reduced ocular perfusion pressure, promoting the loss of retinal ganglion cell death due to ischemia of the optic nerve head (7, 8). However, beyond the MAP level, fluctuations in the 24-h MAP may also destabilize the ocular perfusion pressure due to repetitive and extreme MAP drops over 24-h (9). Approximately 60–80% of these extreme dips in the MAP occur during the day in both normotensive and hypertensive individuals (9), indicating that low MAP is not only limited to nocturnal hypotension. This is critical as the perfusion pressure to the eyes is about 2/3 lower than at the brachial level in the standing or sitting positions—the often diurnal postures (3). In normal physiological conditions, eyes have the ability to maintain their blood flow despite changes in the perfusion pressure by autoregulatory mechanisms (7). However, eyes with glaucoma exhibit irregular autoregulation (7). Therefore, a physiologically lower perfusion pressure coupled with extreme diurnal dips in the MAP would result in glaucomatous eyes experiencing a more intense hypoperfusion state in the optic nerve, resulting in the progression of the disease. Building upon this theory, we aimed this study to test the hypothesis that high variability and dips in the diurnal MAP are associated with OAG damage. We also tested that diurnal MAP dips combined with nocturnal hypotension lead to a more severe OAG damage.

## Materials and methods

### Cohort study

We included patients age ≥ 18 or older with OAG from the database available at the Glaucoma Department, University Hospitals Leuven, Belgium to conduct a retrospective, observational, cohort study. We identified 476 Caucasian patients with OAG followed between 1998 and 2019 who underwent 24-h ambulatory blood pressure monitoring at the Glaucoma Department, University Hospitals Leuven. The assessment of the 24-h blood pressure was performed because patients experienced progression of glaucoma damage despite the intraocular pressure being within the normal range during follow-up. Of 476 patients, a total of 110 patients with primary open-angle glaucoma (POAG) were included in the present study. POAG was defined as glaucoma patients with maximal recorded untreated intraocular pressure level above 21 mm Hg. The Ethics Committee of the University Hospitals Leuven approved the secondary use of the data from the glaucoma patients (registration numbers, S65245 and B32220083510).

### Ophthalmological examination

**FIGURE 1:**
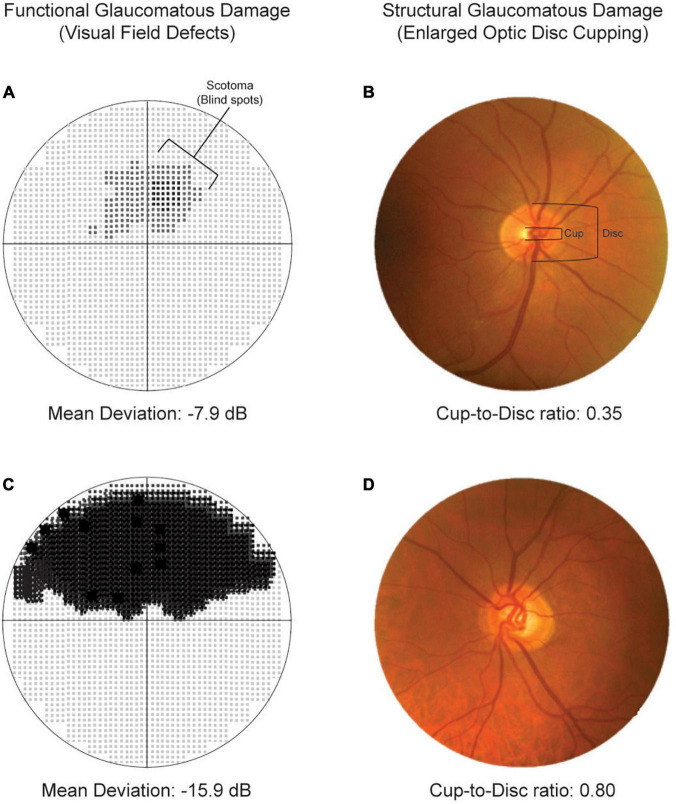
Representation of functional **(left columns)** and structural **(right columns)** glaucomatous damage. Top panels illustrate moderate visual field defects **(A)** and cup-to-disc ratio within the normal range **(B)**. Bottom panels show severe visual field defects **(C)** and enlarged optic disc cupping **(D)**.

The ophthalmic examination was performed by glaucoma specialists, and included measurement of best-corrected visual acuity, biomicroscopy and fundus examination by slit lamp examination and a 90-diopter lens. The intraocular pressure was measured with Goldmann applanation tonometry. Optic disc cupping was the cup-to-disc ratio estimated by glaucoma expert visual rating during the fundus examination. The optic nerve head and the retinal fiber layer were examined by Heidelberg Retinal Tomography (HRT3^®^) or Optical Coherence Tomography Spectralis^®^ (Heidelberg Engineering GmbH, Heidelberg, Germany). The visual field was tested using the Humphrey Visual Field Analyzer HFA3^®^ (Carl Zeiss Meditec AG, Jena, Germany) or the Octopus 300/900 system^®^ (Haag-Streit AG, Switzerland). Glaucoma was diagnosed following the 5th European Glaucoma Society Guidelines, as a significant optic nerve rim and retinal nerve fiber layer thinning with congruent visual field defects. Figure 1 illustrates examples of functional and structural glaucomatous damage. Functional glaucoma damage was derived from the visual field test expressed as mean deviation (decibels, dB). Structural glaucomatous damage (optic disc cupping) was defined as cup-to-disc ratio (1, 10).

### Blood pressure measurements

Office blood pressure was the average of three readings at the initiation of the ambulatory blood pressure recordings in the patients enrolled at the Leuven Glaucoma Department. The ambulatory blood pressure monitoring was performed with validated oscillometric recorders (Mobil-O-Graph devices) (11). The programmed intervals between readings ranged from 15 min (from 06hh00 to 00h00) during daytime and from 30 min (from 00h00 to 06h00) at night (Supplementary Table 1). The within-participant 24-h MAP was a time-weighted average, giving a weight to each individual reading proportional to the interval to the previous reading. Diurnal and nocturnal MAP hypertension were a MAP equal or higher than 96 and 82 mm Hg; respectively (12). As no current operative thresholds are available to define hypotension, we used the within-individual lowest 10th percentile of the diurnal and nocturnal MAP distribution to define diurnal and nocturnal hypotension. We used the variability independent of the mean (VIM) index to quantify the diurnal and nocturnal MAP variability measurements. VIM was calculated as the within-participant standard deviation divided by the mean to the power *x* and multiplied by the population mean to the power *x*. The power *x* was obtained by fitting a curve through a plot of the standard deviation against the mean, using the model: standard deviation = a × *mea*^*nx*^, where x was derived by non-linear regression analysis. The value of x so obtained was 1.05 and 0.77 for estimating diurnal and nocturnal VIMmap. To study extreme dips in MAP, the five diurnal and three nocturnal readings with the largest drops compared to the previous reading in individual MAP readings were selected for further analysis—the time elapsed in between was used to quantify the duration of dips/blips. To identify decrease in the nocturnal MAP levels in relation to diurnal MAP levels, we estimated the night-to-day ratio (13). Night-to-day ratios ≤0.80 indicated an extreme dipping in the nighttime MAP compared to daytime, >0.80 to ≤0.90 indicated a normal physiological nocturnal MAP dipping, and >0.90 indicated non-dipping.

### Statistical analysis

For database management and statistical analysis, we used SAS software, version 9.4, maintenance level 5. Baseline characteristics were reported as arithmetic mean with standard deviation for continuous variables with normal distribution, or median with interquartile ranges for non-parametric continuous variables. Categorical data was reported as frequency and percentage (%). Our outcome was glaucoma progression assessed as longitudinal changes in the (i) mean deviation, expressed in decibels (dB) or (ii) optic disc cupping assessed as cup-to-disc ratio. We conducted unadjusted and adjusted linear mixed models to evaluate the relationship between glaucoma progression and ambulatory MAP measurements. Adjusted models accounted for variables with biological relevance and additionally accounted for the time difference between the baseline visual field test/fundus examination and ambulatory blood pressure monitoring. Supplementary analysis included fully-adjusted models additionally accounted for the diurnal or nocturnal MAP level. In linear mixed models, we introduced a random-effect that accounted for clustering of the observations within participants, that is, accounting for individuals’ initial mean deviation or cup-to-disc ratio instead of the sample average – this was done while accounting for correlation between eyes. Including follow-up time as an intercept in the random statement allows us to construct longitudinal linear mixed models (14, 15). We plotted the progression of glaucoma damage across categories of variability and dips in the diurnal and nocturnal MAP by deriving from mixed modeling the predicted longitudinal mean deviation and cup-to-disc ratio across the categories. We also investigated the contribution of changes in (i) diurnal MAP variability and dips and (ii) nocturnal MAP level in relation to glaucoma progression. Significance was a 2-tailed α-level of ≤ 0.05.

## Results

### Cohort characteristics

The mean age was 68.5 years, and 40.9% (*n* = 45) patients with POAG were women (Table 1). The prevalence of office, diurnal, and nocturnal MAP hypertension was 83.6, 62.3, and 69.1%, respectively, and 6.4% were on antihypertensive medication. Supplementary Table 2 contains the list of medications for lowering the intraocular pressure or systemic blood pressure levels. The rates of current smoking, drinking habits, obesity, diabetes mellitus, and previous cardiovascular diseases ranged from 2.7 to 20.9%. The averaged maximum untreated intraocular pressure was 24 mm Hg—the baseline intraocular pressure was 14 mm Hg. A total of 46 (41.8%) and 40 (36.4%) POAG patients were on eye drops medications or had surgical interventions to lower the intraocular pressure. With a median follow-up time of 9 years, the median number of visual field tests was 11 (interquartile range, 6–20 tests). The median baseline and last mean deviation were −8 and −11 dB. The number of cup-to-disc measures in a median of 5 years was 5.3, and the baseline and last measures were 0.84 and 0.85. The median time between the baseline ambulatory blood pressure monitoring and baseline visual field test was 1.7 years.

**TABLE 1:** Baseline characteristics of glaucoma patients.

Characteristics	Primary-open angle glaucoma (*n* = 110)
**Demographics**	
Women, *n* (%)	45 (40.9)
Age, y	68.5 ± 10.8
**Clinical characteristics**	
Current smoking, *n* (%)	3 (2.7)
Drinking alcohol, *n* (%)	23 (20.9)
Body mass index, kg/m^2^	25.7 ± 3.4
Obesity, *n* (%)	14 (12.7)
Dyslipidemia, *n* (%)	6 (5.4)
Diabetes mellitus, *n* (%)	14 (12.7)
Previous cardiovascular disease	6 (5.4)
Office hypertension, *n* (%)	92 (83.6)
Office mean arterial pressure, mm Hg	102.0 ± 10.9
Diurnal mean arterial hypertension*, *n* (%)	165 (62.3)
Nocturnal mean arterial hypertension, *n* (%)	183 (69.1)
Anti-hypertensive treatment, *n* (%)	7 (6.4)
**Ophthalmic characteristics**	
Averaged max untreated IOP registered, mm Hg	24 (22, 28)
IOP closest to first visual field test, mm Hg	14 (11, 18)
Eye drops medications, *n* (%)	46 (41.8)
Surgical intervention to lower the IOP, *n* (%)	40 (36.4)
**Ophthalmic outcomes**	
**Functional glaucomatous damage (visual field defects)**	
Number of visual field tests, *n*	11 (6, 20)
Follow-up time, years	9 (4, 12)
Mean deviation at baseline, dB	−8 (−14, −3)
Mean deviation at last follow-up, dB	−11 (−16, −6)
**Structural glaucomatous damage (optic disc cupping)**	
Number of cup-to-disc ratio measures, *n*	5.3 (2.3, 8.4)
Follow-up time, years	5 (3, 8)
Cup-to-disc ratio at baseline, dB	0.84 (0.70, 0.90)
Cup-to-disc ratio at last follow-up, dB	0.85 (0.75, 0.93)

IOP, intraocular pressure; ABPM, ambulatory blood pressure monitoring; dB, decibels. Values are arithmetic mean ± SD or median (interquartile range).

*Diurnal and nocturnal mean arterial hypertension defined as an oscillometric calculated 24-h MAP ≥ 96 or ≥ 82 mm Hg, respectively.

### Ambulatory blood pressure measurements

Table 2 contains the measurements of the diurnal and nocturnal MAP level and variability. Nocturnal MAP level (88.4 vs. 99.5 mm Hg) and variability (8.0 vs. 10.6 mm Hg) were lower than diurnal MAP measurements. Based on the 10th lowest percentile of the distribution, diurnal and nocturnal low MAP were set at < 84 and < 74 mm Hg; respectively. Dips measurements seemed similar between diurnal and nocturnal MAP. The average night-to-day MAP ratio was 0.88 mm Hg; 15.5, 47.3, and 37.3% were extreme dipper, normal dippers, and non-dippers.

**TABLE 2:** Level, variability, and dips in the ambulatory mean arterial pressure during diurnal and nocturnal periods.

Ambulatory mean arterial pressure measurements	Primary-open angle glaucoma (*n* = 110)
**Ambulatory mean arterial pressure**	
**Diurnal measures**	
Average level, mm Hg	99.5 ± 9.8
Low MAP level, < 84 mm Hg	16 (5.4%)
VIM, mm Hg	10.6 ± 2.8
**Extreme dips during daytime**	
Dips minus daytime MAP, mm Hg	76.2 ± 13.5
Dips minus forgoing reading, mm Hg	−16.8 ± 5.5
Ratio dip/forgoing reading, mm Hg	0.84 ± 0.04
**Nocturnal measures**	
Average level, mm Hg	88.4 ± 11.2
Low MAP level, < 74 mm Hg	17 (5.8%)
VIM, mm Hg	8.0 ± 2.9
Night-to-day MAP ratio, mm Hg	0.88 ± 0.07
**Extreme dips during nighttime**	
Dips minus nighttime MAP, mm Hg	82.3 ± 13.1
Dips minus forgoing reading, mm Hg	−17.0 ± 6.1
Ratio dip/forgoing reading, mm Hg	0.83 ± 0.05

POAG, primary open-angle glaucoma; VIM, variability independent of the mean.

### Diurnal variability and dips in mean arterial pressure

**FIGURE 2:**
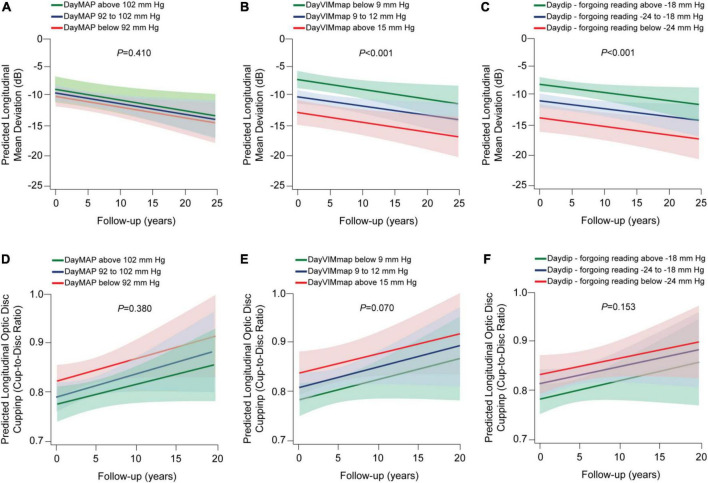
Predicted progression of glaucoma damage across categories of variability and dips in the diurnal mean arterial pressure (map) in patients with primary open-angle glaucoma. VIM refers to variability independent of the mean. We derived the predicted longitudinal mean deviation **(A–C)** and optic disc cupping **(D–F)** from mixed modeling while accounting for sex, age, body mass index, diabetes mellitus, dyslipidemia, smoking habits, in-office intraocular pressure closest to the visual field test, past (max) intraocular pressure registered, eye drops and surgical treatment for lowering the intraocular pressure, use of antihypertensive medication, time-difference between the visual field test and the ambulatory blood pressure monitoring, and the 24-h MAP level. Vertical lines represent the 95% confidence interval. *P*-values are for trend in changes in the mean deviation **(A–C)** and optic disc cupping **(D–F)** across categories.

In univariate adjusted models, diurnal MAP level or low MAP level (below 84 mm Hg) were not associated with the progression of glaucoma damage (*P* ≥ 0.051; Table 3). Every 3 increase in the VIMmap was related to −2.57 dB [95% confident interval (CI), −3.86 to −1.27 dB] changes in the mean deviation (*P* < 0.001) regardless of potential confounders and diurnal (Table 3) or nocturnal (Supplementary Table 3) MAP level. All diurnal measures were significantly associated with progression of glaucoma damage; the estimates ranged from −3.05 dB (95% CI, −4.28 to −1.83; *P* < 0.001) to –1.59 dB (95% CI, −2.63, −0.55; *P* = 0.003). Neither diurnal VIMmap nor dip measures were significantly related to the progression of structural glaucoma damage (Table 3). In Figure 2, the predicted mean deviation was the same across categories of diurnal MAP level (Figure 2A, *P* = 0.380), however, the predicted mean deviation was worse in relation to higher diurnal variability (Figure 2B; *P* < 0.001) and lower MAP dips (Figure 2C; *P* < 0.001). The predicted cup-to-disc ratio was similar across categories of diurnal MAP level (Figure 2D), variability (Figure 2E), and dips (Figure 2F).

**TABLE 3:** Mixed models for the association of progression of glaucoma damage in relation to level, variability, and dips in the diurnal mean arterial pressure in primary open-angle glaucoma.

Diurnal mean arterial pressure measurements	Progression of visual field defects (dB)		Progression of optic disc cupping (cup-to-disc ratio)	
		
	Unadjusted	Adjusted by covariables*	Unadjusted	Adjusted by covariables*
				
	Estimate (95% CI)	Estimate (95% CI)	Estimate (95% CI)	Estimate (95% CI)
**Diurnal measures**				
Average level, −5 mm Hg	−0.43 (−0.94, 0.08)	−0.21 (−0.77, 0.36)	0.01 (−0.01, 0.02)	0.01 (−0.01, 0.02)
Low MAP level, < 84 mm Hg	−1.90 (−8.20, 4.40)	−1.18 (−7.61, 5.25)	−0.05 (−0.17, 0.05)	0.07 (−0.05, 0.19)
VIM, + 3 mm Hg	−2.85 (−4.05, −1.66)^§^	−2.57 (−3.86, −1.27)^§^	0.03 (0.01, 0.05)	0.03 (−0.01, 0.05)
**Extreme dips during daytime**				
Duration of dips, + 30 min	−1.59 (−2.63, −0.55)^‡^	−1.70 (−2.75, −0.58)^‡^	0.01 (−0.01, 0.03)	0.01 (−0.01, 0.03)
Dips minus daytime MAP, −10 mm Hg	−1.67 (−2.45, −0.88)^§^	−2.56 (−3.61, −1.51)^§^	0.02 (0.01, 0.03)^†^	0.02 (−0.01, 0.04)
Dips minus forgoing reading, −6 mm Hg	−2.47 (−3.66, −1.29)^§^	−3.19 (−4.53, −1.84)^§^	0.02 (−0.01, 0.05)	0.03 (0.01, 0.06)^†^
Ratio dip/forgoing reading, −0.05 mm Hg	−3.05 (−4.28, −1.83) ^§^	−3.00 (−4.34, −1.67)^§^	0.03 (0.01, 0.05)^†^	0.02 (−0.01, 0.05)

MAP, mean arterial pressure; VIM, variability independent of the mean. Estimates are association sizes, given with 95% confidence interval (CI), and relate to longitudinal changes in the mean deviation through the follow-up period. For the visual field, negative estimates indicate worsening in the visual field defects, while higher estimates for the cup-to-disc ratio indicate enlargement in the optic disc cupping.

*Mixed models accounted for the within-participant and eye side clustering, and were adjusted for sex, age, body mass index, diabetes mellitus, dyslipidemia, smoking habits, in-office intraocular pressure closest to the visual field test, past untreated (max) intraocular pressure, eye drops and surgical treatment for lowering the intraocular pressure, use of antihypertensive medication, follow-up time, and time-difference between the visual field test and the ambulatory blood pressure monitoring. Models additionally accounted for nocturnal MAP level are shown in Supplementary Table 3.

^†^*P* ≤ 0.05; ^‡^*P* ≤ 0.01; ^§^*P* ≤ 0.001.

### Nocturnal variability and dips in mean arterial pressure

**FIGURE 3:**
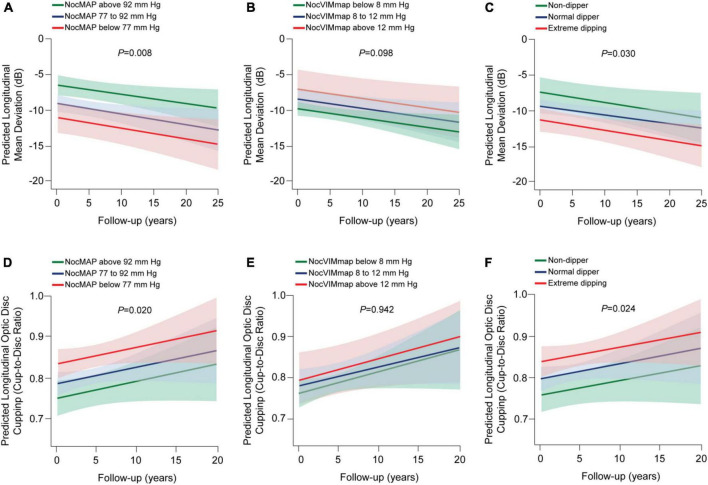
Predicted progression of glaucoma damage across categories of variability and dips in the nocturnal mean arterial pressure (MAP) in patients with primary open-angle glaucoma. Noc refers to nocturnal; VIM, variability independent of the mean. We derived the predicted longitudinal mean deviation **(A–C)** and optic disc cupping **(D–F)** from mixed modeling while accounting for sex, age, body mass index, diabetes mellitus, dyslipidemia, smoking habits, in-office intraocular pressure closest to the visual field test, past (max) intraocular pressure registered, eye drops and surgical treatment for lowering the intraocular pressure, use of antihypertensive medication, time-difference between the visual field test and the ambulatory blood pressure monitoring, and the 24-h MAP level. Vertical lines represent the 95% confidence interval. *P-*values are for trend in changes in the mean deviation **(A–C)** and optic disc cupping **(D–F)** across categories. Dip measures did not associate with glaucoma progression (*P* ≥ 0.254).

Contrary to diurnal, lower MAP level was associated with −0.71 dB changes in the mean deviation (Table 4, 95% CI, −1.24 to −0.17; *P* = 0.003) and 0.01 larger optic disc cupping (95% CI, 0.01–0.02; *P* = 0.014). Patients with a nocturnal MAP level below 74 mm Hg had a −7.54 dB change in the mean deviation (*P* = 0.005). Every 0.05 mm Hg decrease in the night-to-day ratio related to −0.98 dB change in the mean deviation (95% CI, −1.72 to −0.24; *P* = 0.006) and 0.02 larger cup-to-disc ratio (95% CI, 0.01–0.03; *P* = 0.012). In a categorical analysis of the night-to-day ratio, we did not observe a significant association of extreme dipping with glaucoma damage (*P* ≥ 0.051). Nocturnal VIMmap was not associated with glaucoma damage (*P* = 0.054). Only the duration of the nocturnal dips and dips minus the nocturnal MAP level were significantly associated with changes in the mean deviation—the estimates were −1.59 and −1.37 dB; respectively. The other nocturnal dip measures were not statistically related to glaucoma damage (*P* ≥ 0.224). Figure 3 displays the predicted longitudinal mean deviation and optic disc cupping in relation to categories in the nocturnal MAP level, variability, night-to-day ratio, and dips. Progression of the glaucoma damage worsened across categories of MAP level (Figure 3A; *P* = 0.008) and night-to-day ratio (Figure 3C; *P* = 0.030), but did not worsen for variability or dips categories (Figures 3B,D–F). Adjustment by diurnal MAP level did not modify the mentioned findings (Supplementary Table 4).

**TABLE 4:** Mixed models for the association of progression of glaucoma damage in relation to level, variability, and dips in the nocturnal mean arterial pressure in primary open-angle glaucoma.

Nocturnal mean arterial pressure measurements	Progression of visual field defects (dB)		Progression of optic disc cupping (cup-to-disc ratio)	
		
	Unadjusted	Adjusted by covariables*	Unadjusted	Adjusted by covariables*
				
	Estimate (95% CI)	Estimate (95% CI)	Estimate (95% CI)	Estimate (95% CI)
**Nocturnal measures**				
Average level, −5 mm Hg	−0.50 (−0.97, −0.03)^‡^	−0.71 (−1.24, −0.17)^‡^	0.01 (0.01, 0.02)^†^	0.01 (0.01, 0.02)^†^
Low MAP level, < 74 mm Hg	−7.23 (−12.1, −2.40)^‡^	−7.54 (−12.6, −2.52)^‡^	0.04 (−0.05, 0.13)	−0.04 (−0.14, 0.06)
VIM, + 3 mm Hg	0.45 (−0.59, 1.50)	0.91 (−0.17, 1.99)	0.01 (−0.02, 0.02)	−0.01 (−0.02, 0.02)
Night−to−day ratio, −0.05 mm Hg	−0.96 (−1.70, −0.21)^‡^	−0.98 (−1.72, −0.24)^‡^	0.01 (−0.01, 0.02)	0.02 (0.01, 0.03)^†^
**Dipping status,**				
Normal dipping	Reference group	Reference group	Reference group	
Extreme dipping	−2.25 (−5.27, 0.78)	−3.05 (−6.19, 0.08)	−0.02 (−0.07, 0.03)	−0.03 (−0.09, 0.03)
Reverse dipping	−1.15 (−3.30, 0.99)	1.04 (−1.26, 3.33)	0.01 (−0.04, 0.04)	0.02 (−0.03, 0.07)
**Extreme dips during nighttime**				
Duration of dips, + 30 min	−1.70 (−2.93, −0.44)^‡^	−1.52 (−2.83, −0.20)^†^	0.02 (−0.01, 0.04)	0.02 (−0.01, 0.04)
Dips minus nighttime MAP, −10 mm Hg	−1.2 (−1.94, −0.47)^‡^	−0.69 (−1.65, 0.28)	0.01 (−0.01, 0.03)	0.01 (−0.02, 0.02)
Dips minus forgoing reading, −6 mm Hg	−0.31 (−1.21, 0.58)	0.01 (−1.07, 1.07)	0.03 (−0.01, 0.02)	0.01 (−0.02, 0.03)
Ratio dip/forgoing reading, −0.05 mm Hg	−0.91 (−1.83, 0.01)	−0.39 (−1.46, 0.69)	0.01 (−0.01, 0.03)	0.01 (−0.02, 0.02)

MAP, mean arterial pressure; VIM, variability independent of the mean. Estimates are association sizes, given with 95% confidence interval (CI), and relate to longitudinal changes in the mean deviation through the follow-up period. For the visual field, negative estimates indicate worsening in the visual field defects, while higher estimates for the cup-to-disc ratio indicate enlargement in the optic disc cupping.

*Mixed models accounted for the within-participant and eye side clustering, and were adjusted for sex, age, body mass index, diabetes mellitus, dyslipidemia, smoking habits, in-office intraocular pressure closest to the visual field test, past untreated (max) intraocular pressure, eye drops and surgical treatment for lowering the intraocular pressure, use of antihypertensive medication, follow-up time, and time-difference between the visual field test and the ambulatory blood pressure monitoring. Models additionally accounted for diurnal; MAP level are shown in Supplementary Table 4.

^†^*P* ≤ 0.05; ^‡^*P* ≤ 0.01.

### Contribution of diurnal variability and nocturnal hypotension mean arterial pressure to glaucoma

**FIGURE 4:**
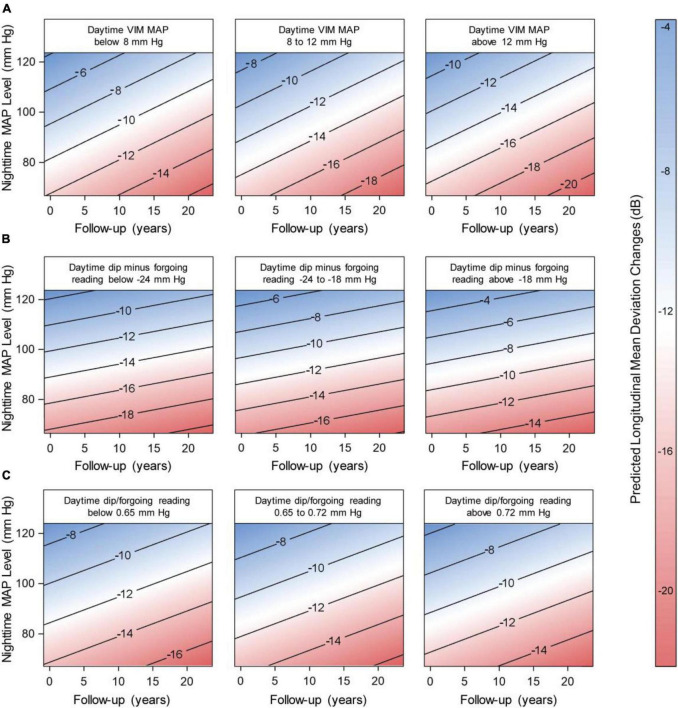
Predicted longitudinal mean deviation in relation to nocturnal mean arterial pressure (MAP) level along with variability and dips in the daytime map. The numbers within the contour graphics represent the predicted changes in the mean deviation (dB) during follow-up time. The blue-white-red bar indicates the severity of the changes, where blue represents the smaller change and red the largest. We derived the predicted longitudinal changes from mixed modeling while accounting for sex, age, body mass index, diabetes mellitus, dyslipidemia, smoking habits, in-office intraocular pressure closest to the visual field test, past (max) intraocular pressure registered, eye drops and surgical treatment for lowering the intraocular pressure, use of antihypertensive medication, and time-difference between the visual field test and the ambulatory blood pressure monitoring, and by the time-difference between the visual field test and the 24-h intraocular pressure assessment. Vertical lines represent the 95% confidence interval. For **(A)**, the *P*-values of the contribution of nocturnal MAP level and daytime MAPVIM were 0.008 and < 0.001, respectively. The *P*-values of nocturnal MAP level combined with daytime dip measures were < 0.001 for **(B)**, and 0.022 and 0.383 in **(C)**.

Structural glaucoma damage was only associated with low nocturnal MAP, therefore, we plotted in Figure 4 the contribution of diurnal variability and nocturnal MAP to functional glaucomatous damage. Lower nocturnal MAP across tertiles of diurnal VIMmap was associated with worse mean deviation (*P* < 0.009; Figure 4A). The lower the tertiles of dip measures combined with low nocturnal MAP level was associated with worse glaucoma damage (*P* < 0.001; Figure 4B). When we simultaneously analyzed diurnal and nocturnal MAP measures, we observed that both phenotypes independently contributed to the progression of glaucoma damage (Supplementary Table 5). For instance, in model 1, every 5 decrease in the nocturnal MAP level with + 3 increase in diurnal VIMmap were associated with −0.80 dB (95% CI, −1.33 to −0.27; *P* = 0.003) and −2.54 dB (95% CI, −3.80 to −1.28; *P* < 0.001) longitudinal changes in the mean deviation.

## Discussion

Our study results indicate that the diurnal profile of sporadic drops in the MAP during the daytime due to high MAP variability is related to the progression of glaucoma damage rather than the absolute diurnal MAP level. On the contrary, low nocturnal MAP level—rather than variability—was associated with functional and structural glaucomatous damage. The progression of functional glaucomatous damage worsened if nocturnal hypotension was accompanied by high variability or extreme dips in the diurnal MAP.

Our finding that nocturnal hypotension was associated with the progression of functional and structural glaucomatous damage among patients with POAG is in agreement with numerous studies (4–6). Aside from nocturnal hypotension, low office hypotension is also associated with OAG (16–18). Notwithstanding the previous evidence, there is no documentation of the role of diurnal MAP profile in relation to OAG. In our study, we found that high diurnal MAP variability was associated with the progression of glaucomatous damage independently of the average MAP level. Moreover, we observed that repetitive and extreme dips in the MAP were also related to glaucoma damage. Only two studies conducted in the 1990s have investigated diurnal MAP drops but in patients with focal ischemic glaucoma and limited assessment of the diurnal MAP profile (19, 20). Lastly, when we evaluated the contribution of diurnal and nocturnal MAP profiles, we observed that the progression of glaucomatous damage worsened if nocturnal hypotension occurred alongside extreme dips in the diurnal MAP. Our findings suggest that, beyond nocturnal hypotension, the diurnal blood pressure profile provides additional predictive information to assess the risk of glaucoma progression.

The clinical perspective of our findings considers the identification of glaucoma patients experiencing sporadic drops in the diurnal MAP that could lead to reduced ocular perfusion pressure. It is important to differentiate between repetitive drops and diurnal hypotension—the last is more complex to recognize than nocturnal hypotension. The complexity relies on the diurnal MAP level being at the range where autoregulation plateaus are preserved (21). The eyes have autoregulatory mechanisms that respond to changes in the perfusion pressure to maintain blood flow (21). A theoretical range determines to what extent such blood flow is preserved when the perfusion pressure raises or drops (22). Considering our findings, we suggest that the mean diurnal MAP did not decrease below the lower limit where autoregulation is interrupted. Of note, in the two studies relating focal ischemic glaucoma with diurnal MAP drops, the mean diurnal MAP was 93.6 (20) and 96 mm Hg (19), which based on our previously proposed thresholds (12), a diurnal MAP ≥ 94 or 96 mm Hg denote high and MAP hypertension. In our current study, 62.3% of the patients had diurnal MAP hypertension, with an average daytime MAP of 99 mm Hg. In numerous studies on OAG, the diurnal MAP level ranged from 90 to 99 mm Hg (4, 23–28). With this in mind, recognizing diurnal hypotension seems improbable but diurnal sporadic hypotensive episodes might be a novel risk factor for OAG. Moreover, future studies need to clarify whether this autoregulatory range might be decreased in glaucoma patients in accordance with microvascular or endothelial dysfunction.

The perfusion pressure to the eyes is physiologically lower than at the brachial level in the standing and sitting positions (3). This is because of an increased hydrostatic force. Therefore, the estimation of the ocular perfusion pressure corrects MAP by a factor of 2/3 (3, 8). Although individuals’ postural positions are commonly supine or seated during the daytime, it is possible that eyes with glaucoma maintain the blood flow in the presence of such physiologically lower ocular perfusion pressure. However, alongside extreme diurnal MAP dips, the perfusion pressure might sporadically drop below the lower limit where autoregulation is impaired (22), leading to ischemia in the optic nerve head. This hypothesis might support our findings that diurnal, but not nocturnal, MAP variability and repetitive dips relate to glaucoma damage progression. Moreover, we previously reported that when the five largest drops in the MAP over 24-h are identified, about 60–80% of these extreme dips in the MAP occurred during the day in both normotensive and hypertensive individuals (9). A lower perfusion pressure due to postural changes coupled with extreme diurnal dips in the MAP is another clinical aspect to be considered when assessing glaucoma risk.

24-h intraocular pressure was not assessed in our study. Nevertheless, it is important to put in perspective our findings with regards to 24-h intraocular pressure. Patients with glaucoma progression have more diurnal peaks and fluctuations in the intraocular pressure compared to patients without progression (29, 30). Such fluctuations also relate to changes in the ocular perfusion pressure in POAG (31), however, MAP variability can also affect the ocular perfusion pressure (7). Taking our findings into perspective, diurnal peaks in the intraocular pressure combined with diurnal MAP dips would likely result in repetitive drops in the ocular perfusion pressure (2), leading to glaucoma damage progression. On the other hand, as similarly reported in our study, glaucoma also relates to nocturnal hypotension (4–6). It is therefore plausible that nocturnal hypotension occurs with elevated nocturnal intraocular pressure. This is in agreement with previous studies showing that the intraocular pressure is higher during the night due to postural changes during the nighttime (32–34) or early morning peaks because of elevated cortisol levels (35, 36). A persistent reduced ocular perfusion pressure might potentially lead to prolonged ischemia in the optic nerve head during the night. This hypothesis remains untested and future studies will have to face the challenge of measuring nocturnal intraocular pressure, as such tools are not readily available. Despite these limitations, the clinical management of glaucoma should improve by investigating and preventing MAP dips, and tailoring treatment to prevent peaks in the intraocular pressure (37, 38).

### Study limitations

Limitation of our study includes the selection of patients with advanced POAG. The University Hospitals of Leuven is a tertiary referral center and therefore a high proportion of included patients have advanced and progressive glaucoma which limits the generalizability of our findings among patients with moderate glaucoma damage. The retrospective nature of our study and lack of 24-h intraocular pressure are also limitations for interpreting our findings. Finally, the criteria to perform ambulatory blood pressure in glaucoma patients was based on the progression of the disease despite the intraocular pressure being kept within the normal range which might represent a selection bias.

## Conclusion

Progression of glaucoma damage in patients with POAG associates with high variability and extreme dips in the diurnal MAP. Structural glaucomatous damage seems more vulnerable to occur in the presence of nocturnal MAP hypotension. Nowadays evidence focuses on nocturnal hypotension, omitting patients with OAG experiencing repetitive extreme drops in the MAP during the daytime. By implementing 24-h ambulatory blood pressure monitoring, it is possible to identify sporadic diurnal and persistent nocturnal hypotension episodes associated with glaucoma damage. In combination with 24-h intraocular pressure, ambulatory blood pressure monitoring might lead to better stratification of glaucoma risk.

## Data availability statement

The datasets presented in this article are not readily available because due to the restrictions based on privacy regulations, local data sharing laws, and informed consent of the participants, data cannot be made freely available in a public repository. Requests to access the datasets should be directed to IS, ingeborg.stalmans@mac.com.

## Ethics statement

The studies involving human participants were reviewed and approved by the Ethics Committee of the University Hospitals Leuven. The patients/participants provided their written informed consent to make scondary use of the data in this study.

## Author contributions

JM, JVE, GM, LA-A, KVK, IS, and Z-YZ contributed to the conception and design of the study. JM, JVE, DW, KVK, TV, SJ, PV, IS, and Z-YZ contributed to the acquisition of data. JM, JVE, and Z-YZ constructed, organized, and managed the database. JM, JVE, LM, and Z-YZ performed the statistical analysis. JM, JVE, KVK, IS, and Z-YZ wrote the draft and sections of the manuscript. All authors contributed to manuscript revision, read, and approved the submitted version.

## References

[1] Weinreb RN Aung T Medeiros FA. The pathophysiology and treatment of glaucoma. A review. *JAMA.* (2014) 311:1901–11. 10.1001/jama.2014.3192 24825645 PMC4523637

[2] Kim KE Oh S Baek SU Ahn SJ Park KH Jeoung JW. Ocular perfusion pressure and the risk of open-angle glaucoma: systematic review and meta-analysis. *Sci Rep.* (2020) 10:1–12. 10.1038/s41598-020-66914-w 32572072 PMC7308312

[3] Costa VP Harris A Anderson D Stodtmeister R Cremasco F Kergoat H Ocular perfusion pressure in glaucoma. *Acta Ophthalmol.* (2014) 92:e252–66. 10.1111/aos.12298 24238296

[4] Graham SL Drance SM Wijsman K Douglas GR Mikelberg FS. Ambulatory blood pressure monitoring in glaucoma: the nocturnal dip. *Ophthalmology.* (1995) 102:61–9. 10.1016/S0161-6420(95)31053-6 7831043

[5] Melgarejo JD Lee JH Petitto M Yépez JB Murati FA Jin Z Glaucomatous optic neuropathy associated with nocturnal dip in blood pressure: findings from the Maracaibo aging study. *Ophthalmology.* (2018) 125:807–14. 10.1016/j.ophtha.2017.11.029 29310962 PMC5963964

[6] Leeman M Kestelyn P. Glaucoma and blood pressure. *Hypertension.* (2019) 73:944–50. 10.1161/HYPERTENSIONAHA.118.11507 30852921

[7] Cherecheanu AP Garhofer G Schmidl D Werkmeister R Schmetterer L. Ocular perfusion pressure and ocular blood flow in glaucoma. *Curr Opin Pharmacol.* (2013) 13:36–42. 10.1016/j.coph.2012.09.003 23009741 PMC3553552

[8] Van Keer K Breda JB Pinto LA Stalmans I Vandewalle E. Estimating mean ocular perfusion pressure using mean arterial pressure and intraocular pressure. *Invest Ophthalmol Vis Sci.* (2016) 57:2260. 10.1167/iovs.16-19375 27124318

[9] Melgarejo JD Eijgen JV Maestre GE Al-Aswad LA Thijs L Mena LJ Open-angle glaucomatous optic neuropathy is related to dips rather than increases in the mean arterial pressure over 24-H. *Am J Hypertens.* (2022) 35:703–14. 10.1093/ajh/hpac028 35218651 PMC9340631

[10] Tatham AJ Weinreb RN Zangwill LM Liebmann JM Girkin CA Medeiros FA. The relationship between cup-to-disc ratio and estimated number of retinal ganglion cells. *Invest Ophthalmol Vis Sci.* (2013) 54:3205–14. 10.1167/iovs.12-11467 23557744 PMC3648225

[11] Weiss W Gohlisch C Harsch-Gladisch C Tölle M Zidek W van der Giet M. Oscillometric estimation of central blood pressure: validation of the Mobil-O-Graph in comparison with the SphymoCor device. *Blood Press Monit.* (2012) 17:128–31. 10.1097/MBP.0b013e328353ff63 22561735

[12] Melgarejo JD Yang W-Y Thijs L Li Y Asayama K Hansen TW Association of fatal and nonfatal cardiovascular outcomes with 24-hour mean arterial pressure. *Hypertension.* (2021) 77:39–48. 10.1097/01.hjh.0000745264.38412.48 33296250 PMC7720872

[13] Fagard RH Thijs L Staessen JA Clement DL De Buyzere ML De Bacquer DA. Night-day blood pressure ratio and dipping pattern as predictors of death and cardiovascular events in hypertension. *J Hum Hypertens.* (2009) 23:645–53. 10.1038/jhh.2009.9 19225527

[14] Fitzmaurice GM Ravichandran C. A primer in longitudinal data analysis. *Circulation.* (2008) 118:2005–10. 10.1161/CIRCULATIONAHA.107.714618 18981315

[15] Laird NM Ware JH. Random-effects models for longitudinal data. *Biometrics.* (1982) 38:963–74. 10.2307/2529876 7168798

[16] Memarzadeh F Ying-Lai M Chung J Azen SP Varma R Los Angeles Latino Eye Study Group. Blood pressure, perfusion pressure, and open-angle glaucoma: the Los Angeles Latino eye study. *Invest Ophthalmol Vis Sci.* (2010) 51:2872–7. 10.1167/iovs.08-2956 20089880 PMC2891455

[17] Hulsman CAA Vingerling JR Hofman A Witteman JCM de Jong PTVM. Blood pressure, arterial stiffness, and open-angle glaucoma: the Rotterdam study. *Arch Ophthalmol.* (2007) 125:805–12. 10.1001/archopht.125.6.805 17562992

[18] Leske MC Wu SY Hennis A Honkanen R Nemesure B. Risk factors for incident open-angle glaucoma: the Barbados eye studies. *Ophthalmology.* (2008) 115:85–93. 10.1016/j.ophtha.2007.03.017 17629563

[19] Meyer JH Brandi-Dohrn J Funk J. Twenty four hour blood pressure monitoring in normal tension glaucoma. *Br J Ophthalmol.* (1996) 80:864–7. 10.1136/bjo.80.10.864 8976695 PMC505639

[20] Bechetoille A Bresson-Dumont H. Diurnal and nocturnal blood pressure drops in patients with focal ischemic glaucoma. *Graefes Arch Clin Exp Ophthalmol.* (1994) 232:675–9. 10.1007/BF00171383 7843593

[21] Wareham LK Calkins DJ. The neurovascular unit in glaucomatous neurodegeneration. *Front Cell Dev Biol.* (2020) 8:452. 10.3389/fcell.2020.00452 32656207 PMC7325980

[22] Pappelis K Choritz L Jansonius NM. Microcirculatory model predicts blood flow and autoregulation range in the human retina: in vivo investigation with laser speckle flowgraphy. *Am J Physiol Heart Circ Physiol.* (2020) 319:H1253–73. 10.1152/ajpheart.00404.2020 32986964

[23] Kwon J Jo Y Jeong D Shon K Kook MS. Baseline systolic versus diastolic blood pressure dip and subsequent visual field progression in normal-tension glaucoma. *Ophthalmology.* (2019) 126:967–79. 10.1016/j.ophtha.2019.03.001 30853468

[24] Renard E Palombi K Gronfier C Pepin JL Noel C Chiquet C Twenty-four hour (nyctohemeral) rhythm of intraocular pressure and ocular perfusion pressure in normal-tension glaucoma. *Invest Ophthalmol Vis Sci.* (2010) 51:882–9. 10.1167/iovs.09-3668 19684006

[25] Shin JW Jo YH Song MK Won HJ Kook MS. Nocturnal blood pressure dip and parapapillary choroidal microvasculature dropout in normal-tension glaucoma. *Sci Rep.* (2021) 11:1–13. 10.1038/s41598-020-80705-3 33420294 PMC7794393

[26] Yoshikawa T Obayashi K Miyata K Saeki K Ogata N. Increased nighttime blood pressure in patients with glaucoma: cross-sectional analysis of the LIGHT study. *Ophthalmology.* (2019) 126:1366–71. 10.1016/j.ophtha.2019.05.019 31230793

[27] Plange N Kaup M Daneljan L Predel H Remky A Arend O. 24-h blood pressure monitoring in normal tension glaucoma: night-time blood pressure variability. *J Hum Hypertens.* (2006) 20:137–42. 10.1038/sj.jhh.1001959 16239898

[28] Lee J Choi J Jeong D Kim S Kook MS. Relationship between daytime variability of blood pressure or ocular perfusion pressure and glaucomatous visual field progression. *Am J Ophtalmol.* (2015) 160:522–37. 10.1016/j.ajo.2015.05.034 26052089

[29] Asrani S Zeimer R Wilensky J Gieser D Vitale S Lindenmuth K. Large diurnal fluctuations in intraocular pressure are an independent risk factor in patients with glaucoma. *J Glaucoma.* (2000) 9:134–42. 10.1097/00061198-200004000-00002 10782622

[30] Wilensky JT. The role of diurnal pressure measurements in the management of open angle glaucoma. *Curr Opin Ophthalmol.* (2004) 15:90–2. 10.1097/00055735-200404000-00005 15021217

[31] Pemp B Georgopoulos M Vass C Fuchsjaeger-Mayrl G Luksch A Rainer G Diurnal fluctuation of ocular blood flow parameters in patients with primary open-angle glaucoma and healthy subjects. *Br J Ophthalmol.* (2009) 93:486–91. 10.1136/bjo.2008.148676 19029154

[32] Kiuchi T Motoyama Y Oshika T. Relationship of progression of visual field damage to postural changes in intraocular pressure in patients with normal-tension glaucoma. *Ophthalmology.* (2006) 113:2150–5. 10.1016/j.ophtha.2006.06.014 16996611

[33] Evans DW Harris A Garrett M Chung HS Kagemann L. Glaucoma patients demonstrate faulty autoregulation of ocular blood flow during posture change. *Br J Ophthalmol.* (1999) 83:809–13. 10.1136/bjo.83.7.809 10381668 PMC1723099

[34] Hirooka K Shiraga F. Relationship between postural change of the intraocular pressure and visual field loss in primary open-angle glaucoma. *J Glaucoma.* (2003) 12:379–82. 10.1097/00061198-200308000-00015 12897586

[35] Kim JH Caprioli J. Intraocular pressure fluctuation: is it important? *J Ophthalmic Vision Res.* (2018) 13:170. 10.4103/jovr.jovr_35_18 PMC5905311 29719646

[36] Liu JHK Kripke DF Twa MD Hoffman RE Mansberger SL Rex KM Twenty-four-hour pattern of intraocular pressure in the aging population. *Invest Ophthalmol Vis Sci.* (1999) 40:2912–7. 10549652

[37] Hughes E Spry P Diamond J. 24-hour monitoring of intraocular pressure in glaucoma management: a retrospective review. *J Glaucoma.* (2003) 12:232–6. 10.1097/00061198-200306000-00009 12782841

[38] Konstas AG Kahook MY Araie M Katsanos A Quaranta L Rossetti L Diurnal and 24-h intraocular pressures in glaucoma: monitoring strategies and impact on prognosis and treatment. *Adv Ther.* (2018) 35:1775–804. 10.1007/s12325-018-0812-z 30341506 PMC6223998

