# Corrigendum: Progression of functional and structural glaucomatous damage in relation to diurnal and nocturnal dips in mean arterial pressure

**DOI:** 10.3389/fcvm.2024.1432035

**Published:** 2024-06-03

**Authors:** Jesus D. Melgarejo, Jan Van Eijgen, Dongmei Wei, Gladys E. Maestre, Lama A. Al-Aswad, Chia-Te Liao, Luis J. Mena, Thomas Vanassche, Stefan Janssens, Peter Verhamme, Karel Van Keer, Ingeborg Stalmans, Zhen-Yu Zhang

**Affiliations:** ^1^ Research Unit Hypertension and Cardiovascular Epidemiology, KU Leuven Department of Cardiovascular Sciences, Studies Coordinating Centre, Katholieke Universiteit Leuven, Leuven, Belgium; ^2^ Laboratory of Neurosciences, Faculty of Medicine, University of Zulia, Maracaibo, Venezuela; ^3^ Department of Ophthalmology, University Hospitals UZ Leuven, Leuven, Belgium; ^4^ Research Group of Ophthalmology, Department of Neurosciences, Katholieke Universiteit Leuven, Leuven, Belgium; ^5^ Rio Grande Valley Alzheimer’s Disease Resource Center for Minority Aging Research (RGV AD-RCMAR), University of Texas Rio Grande Valley, Brownsville, TX, United States; ^6^ School of Medicine, Institute for Neuroscience, University of Texas Rio Grande Valley, Harlingen, TX, United States; ^7^ Department of Human Genetics, School of Medicine, University of Texas Rio Grande Valley, Brownsville, TX, United States; ^8^ Department of Ophthalmology, New York University (NYU) Grossman School of Medicine, NYU Langone Health, New York, NY, United States; ^9^ Department of Informatics, Universidad Politécnica de Sinaloa, Mazatlán, Mexico; ^10^ KU Leuven Department of Cardiovascular Sciences, Centre for Molecular and Vascular Biology, Katholieke Universiteit Leuven, Leuven, Belgium; ^11^ Division of Cardiology, Department of Internal Medicine, University Hospitals UZ Leuven, Leuven, Belgium

**Keywords:** ambulatory blood pressure monitoring (ABPM), diurnal MAP profile, nocturnal hypotension, glaucoma progression, primary open-angle glaucoma

A Corrigendum on Progression of functional and structural glaucomatous damage in relation to diurnal and nocturnal dips in mean arterial pressure By Melgarejo JD, Van Eijgen J, Wei D, Maestre GE, Al-Aswad LA, Liao C-T, Mena LJ, Vanassche T, Janssens S, Verhamme P, Van Keer K, Stalmans I and Zhang Z-Y. (2022). Front. Cardiovasc. Med 9. doi: 10.3389/fcvm.2022.1024044

In the published article, two author names were incorrectly written as Jan V. Eijgen and Karel V. Keer. The correct spelling is Jan Van Eijgen and Karel Van Keer. The abbreviated form is therefore JVE and KVK instead of JE and KK in the Author Contributions statement.

The authors apologize for this error and state that this does not change the scientific conclusions of the article in any way. The original article has been updated.

