# Supplementary figures and images for: Progression of functional and structural glaucomatous damage in relation to diurnal and nocturnal dips in mean arterial pressure
Source: Front Cardiovasc Med. 2022 Nov 15;9:1024044. doi: 10.3389/fcvm.2022.1024044 (PMC9705350; doi:10.3389/fcvm.2022.1024044)

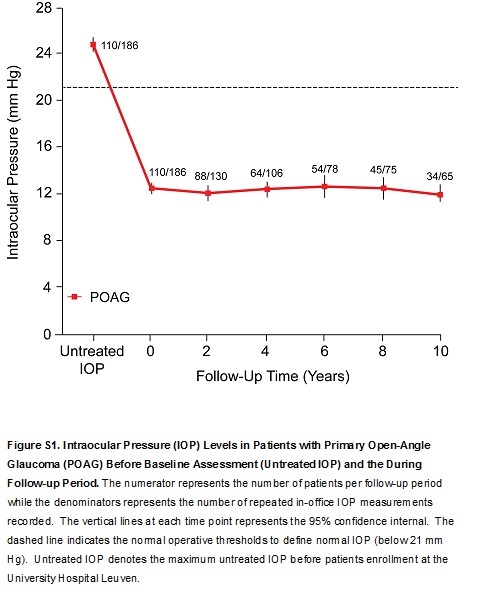

Supplement: Supplementary file 1 [file Image_1.jpg]
