# Supplementary material for: Progression of functional and structural glaucomatous damage in relation to diurnal and nocturnal dips in mean arterial pressure
Source: Front Cardiovasc Med. 2022 Nov 15;9:1024044. doi: 10.3389/fcvm.2022.1024044 (PMC9705350; doi:10.3389/fcvm.2022.1024044)
Supplement: Supplementary file 2 [file Table_1.doc]

**Table S1**

**. Number of Ambulatory Readings by Percentile Ranks**

| **Glaucoma Patients** | **5** | **25** | **50** | **75** | **95** |
| --- | --- | --- | --- | --- | --- |
| Primary Open-Angle Glaucoma (n=110) |  |  |  |  |  |
| 24 hours | 55 | 67 | 71 | 74 | 76 |
| Daytime | 42 | 55 | 58 | 62 | 63 |
| Nighttime | 12 | 13 | 13 | 13 | 13 |

Values are the number of readings corresponding to the 5th, 25th, 50th, 75th and 95th percentiles in 24‑h ambulatory blood pressure recordings. Daytime ranged from 6 AM until 11 PM and nighttime from 11 PM until 6 AM. Readings were programmed at 15‑minute intervals during the day and at 30‑minute intervals.
