# Supplementary material for: Progression of functional and structural glaucomatous damage in relation to diurnal and nocturnal dips in mean arterial pressure
Source: Front Cardiovasc Med. 2022 Nov 15;9:1024044. doi: 10.3389/fcvm.2022.1024044 (PMC9705350; doi:10.3389/fcvm.2022.1024044)
Supplement: Supplementary file 3 [file Table_2.doc]

**Table S2**

**. List of Medications for Lowering the Intraocular Pressure or Systemic Blood Pressure Levels**

| **Medications** | **Primary Open-Angle Glaucoma (n = 110)** |
| --- | --- |
| Ophthalmic medications for lowering IOP |  |
| α-adrenergic agonist | 4 (3.6) |
| β-1 blocker receptors | 0 (0) |
| β-2 blocker receptors | 13 (11.8) |
| Non-selective β-1 and β-2 blocker receptors | 24 (21.8) |
| CA inhibitors + β-2 blocker receptors | 3 (2.7) |
| CA-II inhibitor + β-2 blocker receptors | 0 |
| PPF receptor agonist | 6 (5.4) |
| β-2 blocker receptors + PPF receptor agonist | 4 (3.6) |
| CA-II inhibitor | 0 |
| Number of medications |  |
| 0 | 64 (58.2) |
| 1 | 40 (36.4) |
| 2 | 4 (3.6) |
| 3 | 2 (1.8) |
| Antihypertensive treatments |  |
| ACE-inhibitors | 2 (1.8) |
| AT-1 receptor blockers | 0 |
| β-blockers | 5 (4.6) |
| Diuretics | 0 (0) |
| Calcium channel blockers | 3 (2.7) |
| Number of medications |  |
| 0 | 103 (93.6) |
| 1 | 4 (3.6) |
| 2 | 3 (2.7) |

CA, carbonic anhydrase; IOP, intraocular pressure; PPF, prostanoid prostaglandin-F. *P* values denote the significance of the difference between NTG and POAG patients.
