# Supplementary material for: Progression of functional and structural glaucomatous damage in relation to diurnal and nocturnal dips in mean arterial pressure
Source: Front Cardiovasc Med. 2022 Nov 15;9:1024044. doi: 10.3389/fcvm.2022.1024044 (PMC9705350; doi:10.3389/fcvm.2022.1024044)
Supplement: Supplementary file 4 [file Table_3.doc]

**Table S3**

**. Mixed Models for the Association of Progression of Glaucoma Damage in Relation to Level, Variability, and Dips in the Diurnal Mean Arterial Pressure (MAP) in Primary Open-Angle Glaucoma Additionally Adjusted by Nocturnal MAP Level**

| **Diurnal Mean arterial**  **pressure measurements** | **Fully-Adjusted by**  **Nocturnal MAP Level*** | | | | |
| --- | --- | --- | --- | --- | --- |
|  | **Progression of Visual Field Defects (dB)** | |  | **Progression of Optic Disc Cupping (cup-to-disc ratio)** | |
|  | **Estimate (95% CI)** | ***P* Value** |  | **Estimate (95% CI)** | ***P* value** |
| Diurnal measures |  |  |  |  |  |
| Average level, -5 mm Hg | 0.64 (-1.47, 1.42) | 0.111 |  | -0.01 (-0.02, 0.01) | 0.291 |
| Low MAP level, <84 mm Hg | -0.25 (-6.45, 5.94) | 0.935 |  | 0.09 (-0.03, 0.21) | 0.124 |
| VIM, +3 mm Hg | -2.45 (-3.73, -1.18) | <0.001 |  | 0.02 (-0.01, 0.05) | 0.071 |
| Extreme dips during daytime |  |  |  |  |  |
| Duration of dips, +30 minutes | -1.59 (-2.70, -0.53) | 0.003 |  | 0.01 (-0.01, 0.03) | 0.230 |
| Dips minus daytime MAP, -10 mm Hg | -2.31 (-3.50, -1.11) | <0.001 |  | 0.01 (-0.01, 0.03) | 0.426 |
| Dips minus forgoing reading, -6 mm Hg | -3.06 (-4.40, -1.73) | <0.001 |  | 0.02 (0.01, 0.05) | 0.041 |
| Ratio dip/forgoing reading, -0.05 mm Hg | -2.90 (-4.22, -1.58) | <0.001 |  | 0.02 (-0.01, 0.05) | 0.119 |

MAP, mean arterial pressure; VIM, variability independent of the mean. Estimates are association sizes, given with 95% confidence interval (CI), and relate to longitudinal changes in the mean deviation through the follow-up period. For the visual field, negative estimates indicate worsening in the visual field defects, while higher estimates for the cup-to-disc ratio indicate enlargement in the optic disc cupping.
*Mixed models accounted for the within-participant and eye side clustering, and were adjusted for sex, age, body mass index, diabetes mellitus, dyslipidemia, smoking habits, in-office intraocular pressure closest to the visual field test, past untreated (max) intraocular pressure, eye drops and surgical treatment for lowering the intraocular pressure, use of antihypertensive medication, follow-up time, and time-difference between the visual field test and the ambulatory blood pressure monitoring, and nocturnal MAP level.
