# Supplementary material for: Progression of functional and structural glaucomatous damage in relation to diurnal and nocturnal dips in mean arterial pressure
Source: Front Cardiovasc Med. 2022 Nov 15;9:1024044. doi: 10.3389/fcvm.2022.1024044 (PMC9705350; doi:10.3389/fcvm.2022.1024044)
Supplement: Supplementary file 5 [file Table_4.doc]

**Table S4**

**. Mixed Models for the Association of Progression of Glaucoma Damage in Relation to Level, Variability, and Dips in the Nocturnal Mean Arterial Pressure (MAP) in Primary Open-Angle Glaucoma Additionally Adjusted by Diurnal MAP Level**

| **Nocturnal Mean arterial**  **pressure measurements** | **Fully-Adjusted by**  **Diurnal MAP Level*** | | | | |
| --- | --- | --- | --- | --- | --- |
|  | **Progression of Visual Field Defects (dB)** | |  | **Progression of Optic Disc Cupping (cup-to-disc ratio)** | |
|  | **Estimate (95% CI)** | ***P* Value** |  | **Estimate (95% CI)** | ***P* value** |
| Nocturnal measures |  |  |  |  |  |
| Average level, -5 mm Hg | -1.14 (-1.90, -0.40) | 0.003 |  | 0.02 (0.01, 0.03) | 0.014 |
| Low MAP level, <74 mm Hg | -7.60 (-12.8, -2.29) | 0.005 |  | -0.03 (-0.12, 0.07) | 0.577 |
| VIM, +3 mm Hg | 1.07 (-0.02, 2.16) | 0.054 |  | -0.01 (-0.02, 0.02) | 0.887 |
| Night-to-day ratio, -0.05 mm Hg | -1.07 (-1.82, -0.31 | 0.006 |  | 0.02 (0.01, 0.03) | 0.012 |
| Dipping status, |  |  |  |  |  |
| Normal dipping | Reference group | - |  | Reference group |  |
| Extreme dipping | -3.15 (-6.32, 0.01) | 0.051 |  | -0.03 (-0.09, 0.03) | 0.292 |
| Non-dipping | 1.01 (-1.30, 3.29) | 0.385 |  | -0.02 (-0.03, 0.07) | 0.470 |
| Extreme dips during nighttime |  |  |  |  |  |
| Duration of dips, +30 minutes | -1.59 (-2.90, -0.30) | 0.017 |  | 0.02 (-0.01, 0.04) | 0.206 |
| Dips minus nighttime MAP, -10 mm Hg | -1.37 (-2.44, -0.29) | 0.013 |  | 0.01 (-0.01, 0.03) | 0.514 |
| Dips minus forgoing reading, -6 mm Hg | 0.05 (-1.02, 1.12) | 0.931 |  | 0.01 (-0.02, 0.02) | 0.840 |
| Ratio dip/forgoing reading, -0.05 mm Hg | -0.41 (-1.48, 0.66) | 0.449 |  | 0.01 (-0.02, 0.02) | 0.889 |

MAP, mean arterial pressure; VIM, variability independent of the mean. Estimates are association sizes, given with 95% confidence interval (CI), and relate to longitudinal changes in the mean deviation through the follow-up period. For the visual field, negative estimates indicate worsening in the visual field defects, while higher estimates for the cup-to-disc ratio indicate enlargement in the optic disc cupping.
*Mixed models accounted for the within-participant and eye side clustering, and were adjusted for sex, age, body mass index, diabetes mellitus, dyslipidemia, smoking habits, in-office intraocular pressure closest to the visual field test, past untreated (max) intraocular pressure, eye drops and surgical treatment for lowering the intraocular pressure, use of antihypertensive medication, follow-up time, and time-difference between the visual field test and the ambulatory blood pressure monitoring, and diurnal MAP level.
