# Supplementary material for: Progression of functional and structural glaucomatous damage in relation to diurnal and nocturnal dips in mean arterial pressure
Source: Front Cardiovasc Med. 2022 Nov 15;9:1024044. doi: 10.3389/fcvm.2022.1024044 (PMC9705350; doi:10.3389/fcvm.2022.1024044)
Supplement: Supplementary file 6 [file Table_5.doc]

**Table S5**

**. Adjusted Mixed Models for the Association of Longitudinal Changes in Mean Deviation in Relation to Daytime MAP Variability and Dips Combined with Nighttime MAP Level**

| **Models** | **Longitudinal Changes  in the Mean Deviation (dB)** | |
| --- | --- | --- |
|  | **Estimate (95% CI)*** | ***P* Value** |
| **Model 1** |  |  |
| Nighttime MAP level, -5 mm Hg | -0.80 (-1.33, -0.27) | 0.003 |
| Daytime VIMmap, +3 mm Hg | -2.54 (-3.80, -1.28) | <0.001 |
| **Model 2** |  |  |
| Nighttime MAP level, -5 mm Hg | -0.98 (-1.52, -0.44) | <0.001 |
| Dips minus forgoing reading, -6 mm Hg | -2.14 (-3.37, -0.89) | <0.001 |
| **Model 3** |  |  |
| Nighttime MAP level, -5 mm Hg | -0.70 (-1.22, -0.18) | 0.009 |
| Ratio dip/forgoing reading, -0.05 mm Hg | -2.13 (-3.42, -0.84) | 0.001 |
| **Model 4** |  |  |
| Night-to-day MAP ratio, -0.05 mm Hg | -0.88 (-1.63, -0.13) | 0.021 |
| Daytime VIMmap, +3 mm Hg | -2.32 (-3.60, -1.05) | <0.001 |
| **Model 5** |  |  |
| Night-to-day MAP ratio, -0.05 mm Hg | -0.93 (-1.67, -0.19) | 0.013 |
| Dips minus forgoing reading, -6 mm Hg | -1.39 (-2.60, -0.19) | 0.023 |
| **Model 6** |  |  |
| Night-to-day MAP ratio, -0.05 mm Hg | -0.92 (-1.65, -0.19) | 0.014 |
| Ratio dip/forgoing reading, -0.05 mm Hg | -2.06 (-3.35, -0.77) | 0.002 |

MAP, mean arterial pressure; VIM, variability independent of the mean. Estimates are association sizes, given with 95% confidence interval (CI), and relate to longitudinal changes in the mean deviation through the follow-up period. Negative changes indicate worsening in the visual field test.
*Mixed models accounted for the within-participant and eye side clustering, and were adjusted for sex, age, body mass index, diabetes mellitus, dyslipidemia, smoking habits, in-office IOP closest to the visual field test, past untreated (max) IOP, eye drops and surgical treatment for lowering the IOP, use of antihypertensive medication, follow-up time, and time-difference between the visual field test and the ambulatory BP monitoring.
